# Is Only Clarithromycin Susceptibility Important for the Successful Eradication of *Helicobacter pylori*?

**DOI:** 10.3390/antibiotics9090589

**Published:** 2020-09-09

**Authors:** Young Min Kim, Kyoung Hwa Lee, Jie-Hyun Kim, Soon Young Park, Young Goo Song, Se Yeon Jeon, Hyojin Park

**Affiliations:** 1 Divisions of Gastroenterology, Department of Internal Medicine, Gangnam Severance Hospital, Yonsei University College of Medicine, 211 Eonjuro, Gangnam-gu, Seoul 06273, Korea; mdphant@yuhs.ac (Y.M.K.); sy8503@yuhs.ac (S.Y.J.); hjpark21@yuhs.ac (H.P.); 2 Divisions of Infectious Diseases, Department of Internal Medicine, Gangnam Severance Hospital, Yonsei University College of Medicine, 211 Eonjuro, Gangnam-gu, Seoul 06273, Korea; khlee0309@yuhs.ac (K.H.L.); whwhtnsduddl@naver.com (S.Y.P.)

**Keywords:** *Helicobacter pylori*, eradication rate, antimicrobial susceptibility testing, standard triple therapy, concomitant therapy

## Abstract

Resistance to clarithromycin and other antibiotics included in the eradication regimen, such as amoxicillin and metronidazole, is important for *Helicobacter pylori* (*H. pylori*) eradication. The aim of this study was to investigate the correlation between the results of antimicrobial susceptibility testing and the eradication rate, as well as to understand the importance of antimicrobial susceptibility testing in *H. pylori* eradication. We retrospectively reviewed the electronic medical records of 1862 patients who underwent gastric biopsy for the culture of *H. pylori* during upper endoscopy from March 2015 to June 2019. We tried to find a correlation between the results of the antimicrobial susceptibility testing and the eradication rate in patients who received standard triple or concomitant therapy. A total of 247 patients exhibited positive results for culture and underwent antimicrobial susceptibility testing. Of these, 146 received eradication therapy, with follow-up tests after treatment. In the standard triple therapy, patients who were susceptible to both clarithromycin and amoxicillin exhibited significantly higher eradication rates (85.9%) than those susceptible to clarithromycin and resistant to amoxicillin (75.0%) or those resistant to clarithromycin and susceptible to amoxicillin (44.4%) (*p* = 0.013). In the concomitant therapy, patients who were susceptible to both clarithromycin and metronidazole had significantly higher eradication rates (96.3%) than those susceptible to clarithromycin and resistant to metronidazole (88.9%) or those resistant to clarithromycin and susceptible to metronidazole (50.0%) (*p* = 0.016). There was a correlation between the results of antimicrobial susceptibility testing and the eradication rate for *H. pylori*. In addition to clarithromycin, susceptibility to amoxicillin and metronidazole is also important for the successful eradication of *H. pylori*.

## 1. Introduction

*Helicobacter pylori* (*H. pylori*), which is a Gram-negative and spiral-shaped bacterium, is associated with gastric diseases such as chronic gastritis, peptic ulcers, and gastric mucosa-associated lymphoid tissue (MALT) lymphoma. Previous studies had shown *H. pylori* to be an independent risk factor for gastric carcinogenesis [1,2,3]. Not only gastric cancer but also precancerous lesions such as intestinal metaplasia and atrophic gastritis were associated with *H. pylori* infection. Therefore, eradication therapy is important for preventing the progression of precancerous lesions into gastric cancer.

A previous meta-analysis reported that the prevalence of *H. pylori* varied widely according to geographic area [4]. The region with the highest prevalence of *H. pylori* was Africa (70.1%), with Oceania demonstrating the lowest prevalence (24.4%). The prevalence was over 50 % in Eastern Asian countries such as Korea.

In Korea, the eradication rate for standard triple therapy, which is considered the first-line treatment, decreased by 76.4% in 2014 [5,6]. This unsatisfactory eradication rate was caused by several factors including antimicrobial resistance and poor medication compliance [7,8]. Among these factors, antimicrobial resistance is the main problem that prevents the successful eradication of *H. pylori*. Specifically, a recent study showed that the resistance to clarithromycin is approximately 30% [9], and a high resistance rate is one of the primary reasons for failure for *H. pylori* eradication.

Recent Korean guidelines regarding the treatment of *H. pylori* infections (updated in 2013) emphasized empiric therapies such as standard triple therapy and bismuth-based quadruple therapy, rather than tailored therapy based on antimicrobial susceptibility testing [6]. *H. pylori* culture and antimicrobial susceptibility testing are difficult to routinely perform prior to treatment in clinical practice, mainly because they are time consuming and costly, and require difficult conditions [10]. There are some established methods for detecting clarithromycin resistance such as dual priming oligonucleotide–polymerase chain reaction and dual-labeled peptide nucleic acid probes [11,12], but these methods are not widely used in clinical practice.

However, the Maastricht V/Florence Consensus emphasized the importance of antimicrobial susceptibility testing before treatment [13]. According to this consensus, when the rate of resistance to clarithromycin in the region is more than 15%, antimicrobial susceptibility testing before standard triple therapy is recommended. This is more strict than the 15–20% threshold in the previous version (Maastricht IV/Florence Consensus) [14].

Because antimicrobial resistance for *H. pylori* is challenging phenomenon for successful eradication globally, there have been several studies on the antimicrobial susceptibility of *H. pylori* strains [15,16]. However, studies comparing in vitro antimicrobial susceptibility and the results of eradication in vivo have been scarce. Therefore, the aim of this study was to investigate the correlation between antimicrobial susceptibility testing and the eradication rate. Moreover, since the eradication regimen for *H. pylori* consists of multiple antibiotics such as amoxicillin, clarithromycin, metronidazole, and tetracycline [6,17], we focused on individual antibiotics consisting of two regimens and further explored the importance of antimicrobial susceptibility testing for *H. pylori* eradication.

## 2. Materials and Methods

### 2.1. Study Design and Population

We retrospectively reviewed the electronic medical records of 1862 patients who underwent gastric biopsy for *H. pylori* culture during upper endoscopy at the Gangnam Severance Hospital, Seoul, Korea, from March 2015 to June 2019. Patients with positive results for *H. pylori* culture who underwent antimicrobial susceptibility testing were enrolled in this study. All the enrolled patients were Korean and were over 18 years of age.

The study protocol conformed to the ethical guidelines of the World Medical Association Declaration of Helsinki and was approved by the Institutional Review Board of the Gangnam Severance Hospital (3-2015-0193). Informed consent was not required, as this study was a retrospective analysis of existing administrative and clinical data.

### 2.2. Data Collection

We retrospectively collected the data of enrolled patients, which included demographic, clinical, and laboratory parameters. The demographic data included age, sex, height, body weight, body mass index (BMI), and personal and medical history. The clinical data included the eradication regimen for *H. pylori*, medication compliance, and follow-up tests such as the urea breath test (UBT) and rapid urease test (RUT). We checked patient compliance by retrospectively reviewing patient medical records. To ascertain compliance, patients were required to bring their remaining pills after treatment, which were counted. The laboratory data included antimicrobial susceptibility testing for antibiotics included in the eradication regimen.

### 2.3. Isolation of H. pylori Strains and Growth Conditions

We collected gastric biopsy specimens during upper endoscopy. Biopsy specimens from antra and corpora were placed in a transport medium. These specimens were seeded in egg yolk emulsion (EYE) agar plates (Yuhan LabTech, Seoul, Korea). The EYE agar consisted of Columbia agar, 43.82 μg/mL; EYE, 112.36 μL/mL; IsoVitaleX, 11.23 μL/mL; and 2,3,5-triphenyltetrazolium chloride, 45.0 μg/mL (for colony staining). The plates were subsequently stored in a multigas incubator (microaerophilic atmosphere: 10% carbon dioxide, 5% oxygen, and 85% nitrogen at 37 °C) for 3–7 days. If *H. pylori* was not isolated after 7 days of incubation, the plates were incubated for 3 more days. *H. pylori* isolates were identified on the basis of colony morphology and were confirmed by matrix-assisted laser desorption/ ionization-time-of-fight mass spectrometry (MALDI-TOF) using the Microflex LT system (Bruker Daltonics, Bremen, Germany). The measured profiles were compared to a profile hosted on a database using the MALDI Biotyper 3.1 software (Bruker Daltonics). The colonies were re-identified by subculture analysis.

### 2.4. Antimicrobial Susceptibility Testing

We performed antimicrobial susceptibility testing for five antibiotics, amoxicillin, clarithromycin, metronidazole, tetracycline, and levofloxacin, which are all prescribed for *H. pylori* eradication. Susceptibility to these antibiotics was determined by the Epsilometer test (E-test) [18]. The in vitro minimum inhibitory concentrations (MICs) of the five antibiotics against the *H. pylori* clinical isolates were defined by the points of intersection of the inhibitory zones with the strips. To determine susceptibility, we adopted the European Committee on Antimicrobial Susceptibility Testing (EUCAST) guidelines [19]. The MIC breakpoints for amoxicillin, clarithromycin, metronidazole, tetracycline, and levofloxacin were >0.125, >0.5, >8, >1, and >1 mg/L, respectively. Multidrug resistance was defined as resistance against two or more of the antibiotics evaluated.

### 2.5. Eradication Therapy for H. pylori

It takes a long time to identify the results of antimicrobial susceptibility testing after gastric biopsy. Therefore, most of the patients in our hospital received empirical therapy for eradication. Moreover, all the patients enrolled in this study received empirical therapy and had no history of eradication therapy. The eradication regimen in this study included standard triple therapy, concomitant therapy, and bismuth-containing quadruple therapy. Standard triple therapy involved the use of a proton-pump inhibitor (PPI), amoxicillin, and clarithromycin. Concomitant therapy consisted of PPI, amoxicillin, clarithromycin, and metronidazole. Bismuth-containing quadruple therapy consisted of PPI, bismuth, metronidazole, and tetracycline. Patients who received eradication therapy underwent follow-up tests to evaluate the success of the eradication.

### 2.6. Statistical Analysis

Continuous variable values are reported as the mean ± standard deviation, and categorical variable values are reported as the number and percentage. We used the chi-square test or Fisher’s exact test to compare the results of the antimicrobial susceptibility testing to the eradication rates in patients who received standard triple therapy or concomitant therapy. Statistical analysis was performed using SPSS version 23.0 (IBM Corp., Armonk, NY, USA). A two-tailed *p*-value < 0.05 was considered statistically significant.

## 3. Results

### 3.1. Flow Chart and Baseline Characteristics

**Figure 1:**
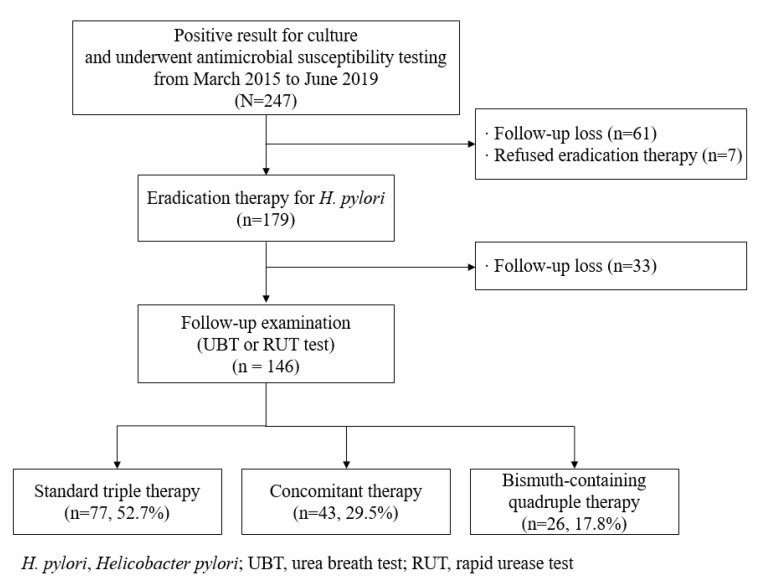
Flow chart for the enrolled patients.

Figure 1 shows a flow chart of this study. Of 247 patients, 61 were lost during follow-up and 7 refused eradication therapy. We retrospectively reviewed 179 patients who received empirical eradication therapy and had no history of eradication. Among these 179 patients, 146 underwent follow-up examinations such as UBT or RUT. Regarding compliance, all 146 patients took more than 80% of their prescribed medication. In addition, 77 patients received standard triple therapy, 43 received concomitant therapy, and 26 received bismuth-containing quadruple therapy as a first-line therapy.

Of the 247 patients, 106 (42.9%) were men, and the mean age was 55.4 ± 11.8 years (Table 1). The average BMI was 23.3 ± 3.4 kg/m^2^. Approximately 18.2% of patients were current smokers, and 41.7% had alcohol consumption histories. Approximately 20.2% and 9.7% of the patients had diabetes mellitus and hypertension, respectively. A total of 223 patients (90.3%) had a positive result for RUT.

### 3.2. Results of Antimicrobial Susceptibility Testing

**Figure 2:**
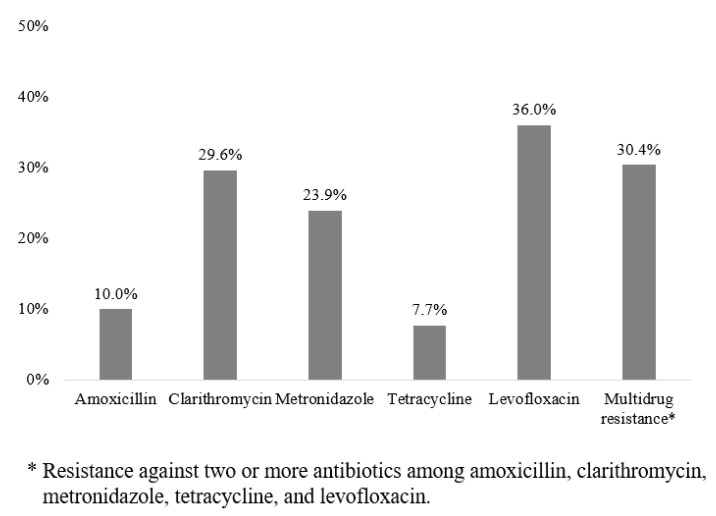
The antimicrobial resistance pattern of the *Helicobacter pylori* isolates (*n* = 247).

Figure 2 shows the antimicrobial resistance pattern of the *H. pylori* strains in this study. The rates of resistance to amoxicillin, clarithromycin, and metronidazole were 10.1%, 29.6%, and 23.9%, respectively. Furthermore, the rates of resistance to tetracycline and levofloxacin were 7.7% and 36.0%, respectively. A total of 75 strains (30.4%) demonstrated multidrug resistance (5 for five drugs, 2 for four drugs, 15 for three drugs, and 53 for two drugs).

### 3.3. Eradication Rate for H. pylori

In this study, the total eradication rate was 86.3%. Among 77 patients who received standard triple therapy, 62 had negative results in follow-up tests (eradication rate: 80.5%). Of the 43 patients who received concomitant therapy, 38 achieved successful eradication (eradication rate: 86.4%). All 26 patients who received bismuth-containing quadruple therapy achieved successful eradication (eradication rate: 100.0%).

### 3.4. Comparison between Antimicrobial Susceptibility Testing and Eradication Rate

**Figure 3:**
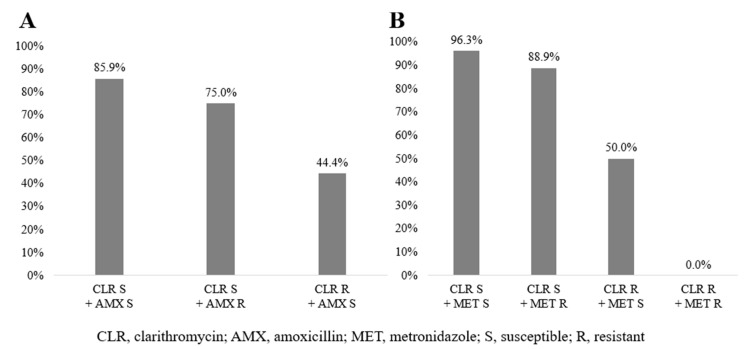
Comparison between antimicrobial susceptibility testing and eradication rate in standard triple therapy (*n* = 77, **A**) and concomitant therapy (*n* = 43, **B**).

In standard triple therapy, patients who were susceptible to both clarithromycin and amoxicillin had an 85.9% eradication rate. Patients who were susceptible to clarithromycin but resistant to amoxicillin had a 75.0% eradication rate. Around 44.4% of the patients who were susceptible to amoxicillin but resistant to clarithromycin achieved successful eradication (Figure 3A). This difference in eradication rate was statistically significant (*p* = 0.013).

Figure 3B shows the results of antimicrobial susceptibility testing and the eradication rate with concomitant therapy. For patients susceptible to both clarithromycin and metronidazole, the eradication rate was 96.3%. Patients who were susceptible to clarithromycin but resistant to metronidazole had an 88.9% eradication rate. Fifty percent of the patients who were susceptible to metronidazole but resistant to clarithromycin achieved successful eradication. One patient who was resistant to both clarithromycin and metronidazole had eradication failure. This result showed a significant difference (*p* = 0.016). A comparison between the susceptibility status for all the antibiotics included in the regimen (amoxicillin, clarithromycin, and metronidazole) and the eradication rate is shown in Appendix A.

## 4. Discussion

Previous studies regarding *H. pylori* infection mainly investigated themes such as the results of antimicrobial susceptibility testing and the comparison between empirical and tailored therapy [20,21]. These studies concluded that tailored therapy was superior to empirical therapy [20,22,23], which suggests that antimicrobial susceptibility testing before treatment can be important for successful eradication. However, these studies merely compared the eradication rates between empirical and tailored therapy, without providing significant evidence. To the best of our knowledge, this is the first study to identify the correlation between the susceptibility status for individual antibiotics included in the regimen and the therapeutic outcome for *H. pylori*.

We determined that the rate of resistance to clarithromycin was 29.6%, which is higher than the results from other studies. One recent Korean nationwide study reported 17.8% as the resistance rate for clarithromycin [24]. This difference might be caused by several factors. First, the characteristics of the geographic area might influence this result. Most patients in our study lived in a region with high antibiotic prescription rates. Moreover, clarithromycin is a macrolide antibacterial agent that is used for various infections in addition to *H. pylori* such as respiratory tract and skin infections [25]. Second, the MIC breakpoint is an important factor for this difference. The MIC breakpoint, which was adopted in previous studies, was an inaccurate criterion [24,26,27]. This makes it difficult to compare the resistance rate for clarithromycin or other antibiotics between studies.

Standard triple therapy and concomitant therapy are considered the first-line empirical regimens in Korea in which antibiotics, such as clarithromycin, amoxicillin, and metronidazole, are combined with a PPI. The clarithromycin susceptibility of *H. pylori* is a significant factor for successful eradication. Molecular factors such as 23S rRNA mutation are the main mechanisms underlying this resistance [28,29,30]. Although the precise mechanism underlying the resistance to other antibiotics is unclear, several gene mutations such as *rdxA*, *frxA*, and *fdxB* (associated with metronidazole resistance) and *pbp1A* (associated with amoxicillin resistance) have been reported [31,32,33,34]. We identified the degree of importance for individual antibiotics included in the regimen, which is the strength of this study.

Among 77 patients who received standard triple therapy, 62 exhibited successful eradication (eradication rate: 80.5%). We analyzed the eradication rate based on the results of antimicrobial susceptibility testing for clarithromycin and amoxicillin. We divided the 77 patients into three groups. The first group was susceptible to clarithromycin and amoxicillin, the second group was susceptible to clarithromycin but resistant to amoxicillin, and the third group was resistant to clarithromycin but susceptible to amoxicillin. The eradication rate was the highest in the first group (eradication rate: 85.9%), and the second group had a higher rate than the third group (*p* = 0.013). This suggests that although the two antibiotics are important for eradication, clarithromycin is more effective than amoxicillin.

Next, we analyzed the 43 patients who received concomitant therapy, and the eradication rate was 86.4%. We also divided the 43 patients into three groups. The first group was susceptible to clarithromycin and metronidazole, the second group was susceptible to clarithromycin but resistant to metronidazole, and the third group was resistant to clarithromycin but susceptible to metronidazole. We found that the first group had the best eradication rate (96.3%) and the third group had poorest (50.0%) (*p* = 0.016). This indicates that although the two antibiotics are important for eradication, clarithromycin is more effective than metronidazole.

This study has several meaningful messages for *H. pylori* eradication. First, it emphasizes the importance of antimicrobial susceptibility testing. Although there are some commercial testing kits for susceptibility testing for clarithromycin [11,35,36], they are not widely used. This study demonstrates the necessity of a molecular testing kit. Moreover, this study also emphasizes the importance of susceptibility testing for not only clarithromycin but also other antibiotics such as amoxicillin and metronidazole. Therefore, a susceptibility testing kit for dual antibiotics is also required for successful eradication. Second, we used a precise cutoff value for the MIC by adopting the EUCAST guidelines. The observed resistance rates for antibiotics in *H. pylori* varied from those in previous studies. This variation is associated with several factors, such as the characteristics of the geographic area, method of susceptibility testing, and MIC breakpoint. Among these factors, the unification of the MIC breakpoint is important for comparing the resistance rate across areas and according to the times. There are two international guidelines for antimicrobial susceptibility testing, the EUCAST guidelines and the Clinical and Laboratory Standards Institute (CLSI) guidelines. Regarding *H. pylori*, the MIC breakpoint in the EUCAST guidelines was mentioned above. The MIC breakpoint for clarithromycin is ≥1.0 mg/L, but the other four antibiotics evaluated do not have established breakpoints in the CLSI guidelines [37]. However, the cutoff breakpoint values in previous studies were not exact [24,26,27]. This makes it challenging to evaluate the precise resistance rate. Therefore, this study is important, as it provides a standard resistance rate when compared to other studies using the EUCAST guidelines. Third, our study indicates that multidrug-resistant (MDR) strains, which have resistance against two or more antibiotics, are important problems in successful eradication. The MDR rate was approximately 30.4% in our study. Moreover, in standard triple therapy and concomitant therapy, the eradication rate was low for MDR strains. Recently, there were several studies regarding MDR in various infectious diseases including *H. pylori* infection [38,39,40]. Based on the results of our study, future studies regarding the mechanisms and new antimicrobial agents for MDR strains of *H. pylori* will be needed.

There were several limitations of our study. First, this was a retrospective study with a small sample size of enrolled patients. Therefore, a prospective and large-scale study is warranted to clarify the significance of our findings. Second, this study was not a nationwide study, and there was a selection bias for the geographic area of the enrolled patients. However, we divided the enrolled patients into three groups according to the susceptibility status in each regimen, and compared the eradication rates between the three groups. Despite the small number and limited geographic characteristics of the enrolled patients, we identified the trend of the susceptibility status for each antibiotic by grouping the patients. Therefore, we believe that classifying patients into three groups was partially helpful in overcoming these limitations.

## 5. Conclusions

A correlation was established between the results of in vitro antimicrobial susceptibility testing and the in vivo eradication rate of *H. pylori*. In addition to clarithromycin—known to be an important factor associated with eradication failure—other antibiotics such as amoxicillin and metronidazole—which are included in the eradication regimen—are also important for the successful eradication of *H. pylori*.

## Figures and Tables

**Table 1:** Baseline characteristics of the study population.

Characteristics	All Patients (*n* = 247)
Age (years, mean ± SD)	55.4 ± 11.8
Male (*n*, %)	106 (42.9)
Height (cm, mean ± SD)	163.2 ± 8.4
Weight (kg, mean ± SD)	64.1 ± 12.2
BMI (kg/m^2^, mean ± SD)	23.3 ± 3.4
Current smoker (*n*, %)	45 (18.2)
Alcohol history (*n*, %)	103 (41.7)
Diabetes mellitus (*n*, %)	50 (20.2)
Hypertension (*n*, %)	24 (9.7)
RUT, positive (*n*, %)	223 (90.3)

BMI, body mass index; RUT, rapid urease test.

## Appendix A

**Table A1:** Comparison between antimicrobial susceptibility testing and eradication rate in concomitant therapy (*n* = 43).

Antimicrobial Susceptibility Testing			Number (%) of Successful Eradications
Clarithromycin	Amoxicillin	Metronidazole	
S	S	S	23/24 (95.8)
S	R	S	3/3 (100.0)
S	S	R	8/9 (88.9)
S	R	R	0/0
R	S	S	2/4 (50.0)
R	R	S	1/2 (50.0)
R	S	R	0/0
R	R	R	0/1 (0.0)

S, susceptible; R, resistant.
